# The Impact of Adherence to Prenatal Multiple Micronutrient Supplements on Gestational Weight Gain in Low- and Middle-Income Countries: A Simulation Study

**DOI:** 10.1016/j.cdnut.2026.109505

**Published:** 2026-07-30

**Authors:** Enju Liu, Edie Weller

**Affiliations:** 1 Biostatistics and Research Design Center, Boston Children’s Hospital, Boston, MA, United States; 2 Division of Gastroenterology, Hepatology and Nutrition, Boston Children’s Hospital, Harvard Medical School, Boston, MA, United States; 3 Department of Pediatrics, Harvard Medical School, Boston, MA, United States

**Keywords:** gestational weight gain, adherence, multiple micronutrient supplements, simulation, low- and middle-income countries

## Abstract

**Background:**

Despite the evidence that prenatal nutritional supplements increase gestational weight gain (GWG) and improve birth and pregnancy outcomes in low- and middle-income countries (LMICs), adherence to prenatal nutritional supplements is generally low among pregnant females.

**Objectives:**

The study aimed to evaluate the impact of varying levels of adherence to prenatal multiple micronutrient supplements (MMS) on GWG using a simulation study that was designed based on the published meta-analysis.

**Methods:**

Simulated datasets with different sample sizes (250–4000 participant females) were generated from an assumed model to represent different proportions of females who adhered to their treatment (50%–100%). The performance metrics reported are bias, empirical variance, and mean squared error.

**Results:**

As the adherence level decreased, the average bias and the mean squared error of the estimated percent adequacy of GWG difference between those with and without supplements increased. With 2000 participants, the average bias was 0.66, 0.53, 0.40, 0.24, 0.14 for 50%, 60%, 70%, 80%, and 90% adherence, respectively. This meant that the estimated difference was significantly underestimated with 50% adherence. Results from the simulations varying the sample size and adherence level showed that at each sample size, decreasing the adherence level resulted in larger bias, and at each adherence level, the sample size does not impact the mean bias. These results also demonstrate that even with a large sample size of 4000 participants, the mean bias remains nearly as large as that observed with a smaller sample size when the adherence is low.

**Conclusions:**

Low adherence leads to significant underestimation of the beneficial effect of prenatal MMS on GWG. Increasing the sample size does not remedy this problem. These results highlight the importance of improving adherence to prenatal nutritional implements among pregnant females in LMICs to maximize GWG, reduce low birth weight, and small-for-gestational-age birth.

## Introduction

Pregnant females in low- and middle-income countries (LMICs) are often at higher risk of multiple micronutrient deficiencies because of food insecurity, low dietary diversity, and increased demands of the developing fetus [[1], [2], [3]]. Previous studies have consistently shown that prenatal multiple micronutrient supplements (MMS) decrease risk of low birth weight and small-for-gestational-age birth and particularly benefit infants born to undernourished or anemic females in these settings [4,5]. In response to the accumulating evidence from LMICs on the beneficial effect of MMS during pregnancy on low birth weight, in 2020, the WHO changed its recommendation on MMSs in pregnancy from “not recommended” to “recommended in the context of rigorous research” [6].

Recent meta-analysis has shown that the provision of maternal MMS to pregnant females in LMICs increases gestational weight gain (GWG), a widely used indicator of nutritional adequacy during pregnancy [7]. GWG reflects the accumulation of both fetal and maternal tissue during pregnancy. Approximately 50% of total GWG is attributable to the fetoplacental unit (fetus, placenta, amniotic fluid, and gravid uterus), ∼25% to physiological increases in maternal blood volume, extracellular fluid, and breast tissue, and the remaining 25% to maternal fat stores [8,9]. Consequently, GWG reflects not only maternal nutritional status but also fetal growth and placental development. Improvement in GWG after prenatal MMS may therefore capture changes in both maternal physiological adaptations and fetal growth, consistent with evidence that MMS can reduce risk of low birth weight and small-for-gestational-age birth. Despite the consistent evidence of its beneficial effects, the implementation of such intervention remains challenging in these settings, and adherence levels among pregnant females who received prenatal micronutrient supplements are generally low. Data from Demographic and Health Surveys have indicated that only 8% of pregnant females in LMICs take ≥180 tablets of iron and folic acid supplements during their entire pregnancy [10]. Similar results were reported in a recent study showing that, on average, 65% of pregnant females in sub-Saharan African countries took their iron supplement for 90 or fewer days during their pregnancy [11].

How low adherence influences the beneficial effect of prenatal micronutrient supplements on pregnancy and birth outcomes has been understudied. A recent meta-analysis has shown that no observed benefit effect of MMS on birth weight among females who took <60% of their supplements [12]. In this paper, we evaluate this topic using a simulation approach that is structured to be consistent with the published meta-analysis, with the aim of informing future research and program implementation in the field of nutritional intervention in LMICs.

### Motivating study

The current simulation is based on the context of the recently published meta-analysis, aimed to examine the effects of prenatal MMS, which contain 15 micronutrients including 30 mg iron and 0.4 mg folic acid, on GWG compared with supplements with iron and folic acid only [7]. Specifically, in the meta-analysis, individual data on pill count or intervention uptake of the assigned regimen from each trial were collected, and adherence was assessed by dividing the amount of regimen consumed by the amount distributed to each female during the overall study period. Pregnant females were classified into adherence (if they consumed ≥90% of the assigned regimen) and nonadherence (consumed <90% of the assigned regimen). The primary outcome is the percent adequacy of GWG, which was calculated by dividing the observed GWG at the time of the last weight measurement by the expected GWG for that week of gestation based on the Institute of Medicine recommendations [9], multiplied by 100. This continuous outcome is independent of gestational age at the time of weight measure or delivery and has been used previously [13].

Results from the meta-analysis showed that mean percent adequacy of GWG was 78%, ranging from 60% to 107% across trials, and that pregnant females who received prenatal maternal MMS had a larger percent adequacy of GWG [weighted mean difference (WMD): 0.86%; 95% confidence interval (CI): 0.28%, 1.44%] compared with those in the control arm (received iron and folic acid supplements only). In addition, adherence to the assigned regimen modified the effect of MMS on percent adequacy of GWG. Maternal MMS was associated with larger percentage adequacy of GWG among females with adherence of 90% or more (WMD: 1.4%; 95% CI: 0.6%, 2.1%), but not among females with adherence of <90% (WMD: 0.1%; 95% CI: −0.9%, 1.1%; *P* for interaction = 0.04). On the basis of these results, we were interested in evaluating how varying the proportion of subjects who adhere (defined as taking ≥90% of assigned pills) would influence the estimation of the parameters in the model. To realize this objective, we used the model parameters from the previous meta-analysis as the basis for the formation of our underlying model used in the simulation study. We evaluated the bias of the estimated parameters of the impact of prenatal MMS compared with iron and folic acid only and reported the average bias, mean squared error, and variance.

## Methods

To demonstrate the importance of ensuring pregnant females adhere to prenatal MMS, we conducted a simulation study to evaluate the effect of different adherence levels in the target population. In this simulation study, the outcome (denoted as *Y*) is the mean percent adequacy of GWG; the independent variables are the treatment (denoted as *X*: *X* = 1 for intervention and *X* = 0 for placebo group) and treatment adherence (denoted as *Z*: *Z* = 1 for adherence and *Z* = 0 for nonadherence). In the meta-analysis, the intervention group received prenatal MMS, and the placebo group received iron and folic acid only. Adherence is defined as consuming ≥90% of required supplements. Using data from the motivating study, the model used for the simulation is:

*Yijk*= b0+*b*1∗*Xij*+b2∗*Zik*∗*Xij*

where *i* = 1, 2, …, *n* (number of participants), *j* denotes the 2 treatments, and *k* denotes the 2 adherence categories (≥90% of required supplements taken compared with <90% of required supplements taken). To generate the individual-level data of percent adequacy of GWG for each female, we assume the data follow a normal distribution with an SD of 15.8. The model parameters are selected to be consistent with the observed mean percent adequacy of GWG in the original study which were: *1*) 77.9% (*b*0) in females who received placebo (*Xij* = 0, *Zik* = 0); *2*) 78.0% (*b*0 + *b*1) for females who received MMS but consumed <90% of the supplements (*Xij* = 1, *Zik* = 0); and *3*) 79.3% (*b*0+*b*1+*b*2) for those who received MMS and consumed ≥90% of the supplement (*Xij* = 1, *Zik* = 1). The resulting model parameters from these 3 means are *b*0 = 77.9, *b*1 = 0.1, and *b*2 = 1.3. These values are used to generate the underlying data for each of the simulated data sets. Given that the motivating study was a randomized trial, all simulations assume that 50% of the subjects are randomly assigned to the placebo arm and 50% are randomly assigned to the MMS arm. The simulation parameters that vary in the study are itemized as follows:

(1) Adherence level-proportion of females who adhere to their treatment levels in the MMS arm. Values considered are 50%, 60%, 70%, 80%, 90%, and 100%.
(2) Sample size: 250, 500, 1000, 2000, and 4000 pregnant female participants.

For each combination of parameters, 10,000 simulated data sets are generated. These 10,000 simulated data sets constitute 1 simulation experiment. For each simulated data set, the model specified above is fit to the data, and the parameters are estimated. The performance measures at a simulated data set level include the *1*) bias, which is calculated as the difference between the observed difference in percent adequacy of GWG between MMS and placebo arms for the specific simulated data set and the true difference [which is equal to 1.4% = (79.3% - 77.9%) when all females adhere to their regimen]; *2*) empirical variance of the observed difference; and *3*) mean squared error, which is calculated as the sum of the bias squared and the empirical variance. For each simulation experiment, the average of the bias, empirical variance, and mean squared error are calculated across the 10,000 simulated data sets. Bar graphs and line graphs are used to show the results.

## Results

**FIGURE 1:**
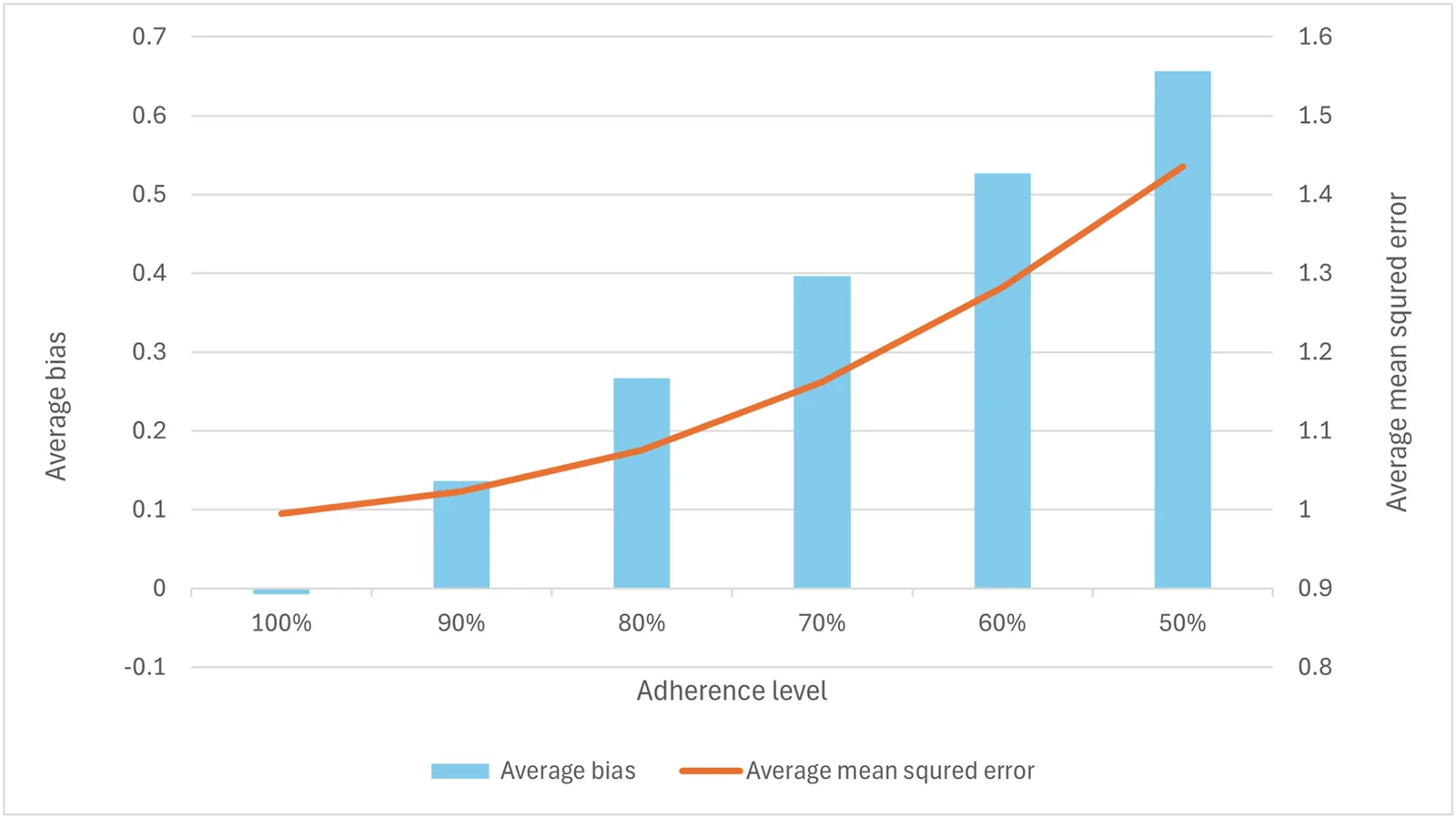
Adherence affects bias and mean squared error. Sample size *n* = 2000.

**FIGURE 2:**
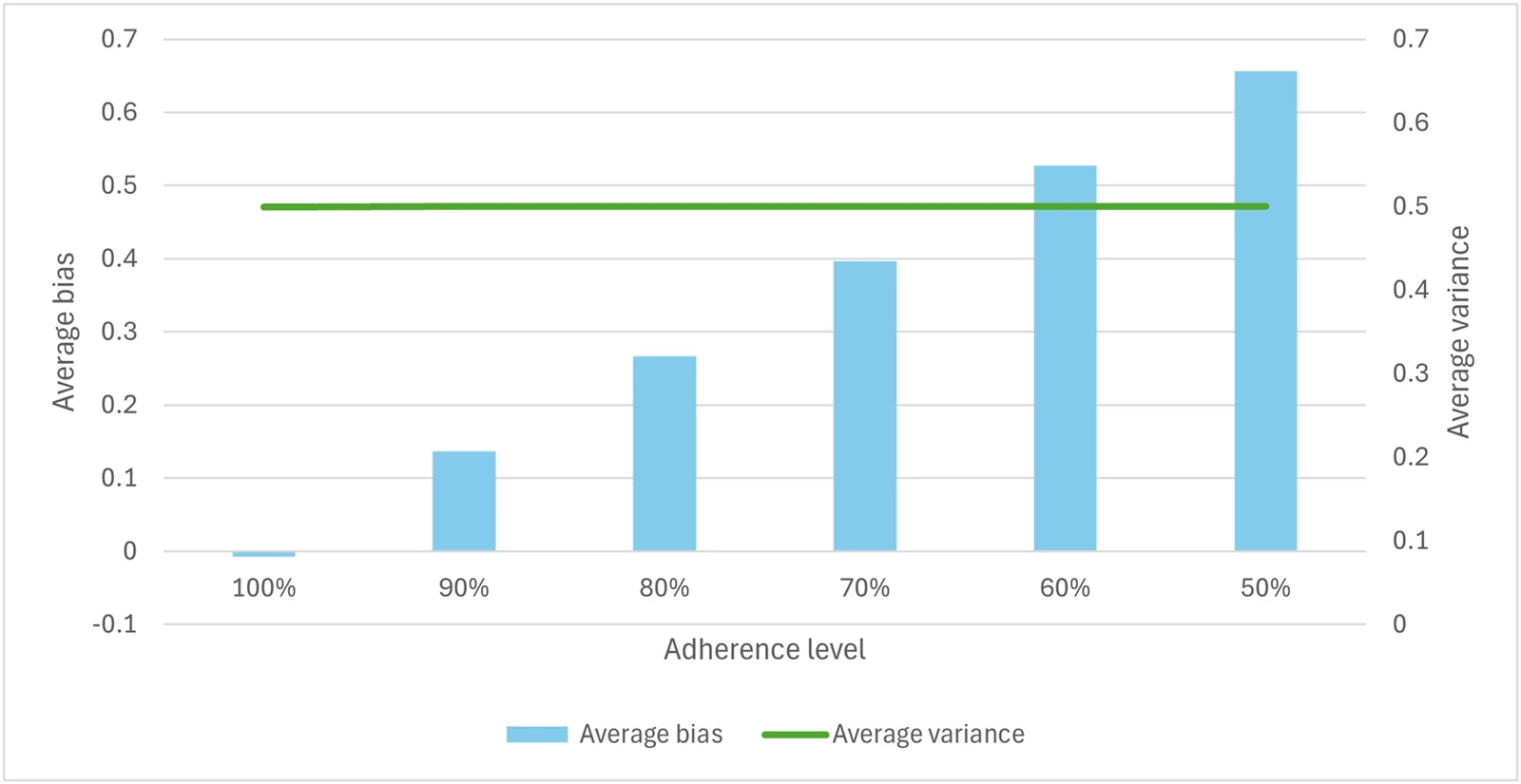
Adherence affects bias but not variance. Sample size *n* = 2000.

Figure 1 shows the relationship among adherence level, bias, and mean squared error with a sample size of 2000 participants in the simulated samples. We can see that the average bias is close to zero, as expected, when the simulated experiment has the highest adherence level (100% of participants adhere). When the adherence level decreases to 90%, the average bias increases to 0.14%, which means the observed effect size reduces on average from the true effect size (1.4% = the true mean difference between MMS and placebo) to 1.26% (e.g., 1.4%–0.14%). If the adherence level further decreases to 50%, the average bias of the 2000 simulated samples increases to 0.66%, which is almost half of the true effect size of 1.4%. Overall, as the adherence level decreases, the bias increases, which results in an underestimate of the true effect of MMS on GWG. We found that the average mean squared error also increases with decreasing the adherence level (Figure 1). This increase is primarily a function of the increase in the bias with decreasing adherence levels because, under a fixed sample size, the empirical variance of the estimated mean difference in GWG percent adequacy does not change as the adherence levels decrease (Figure 2).

**FIGURE 3:**
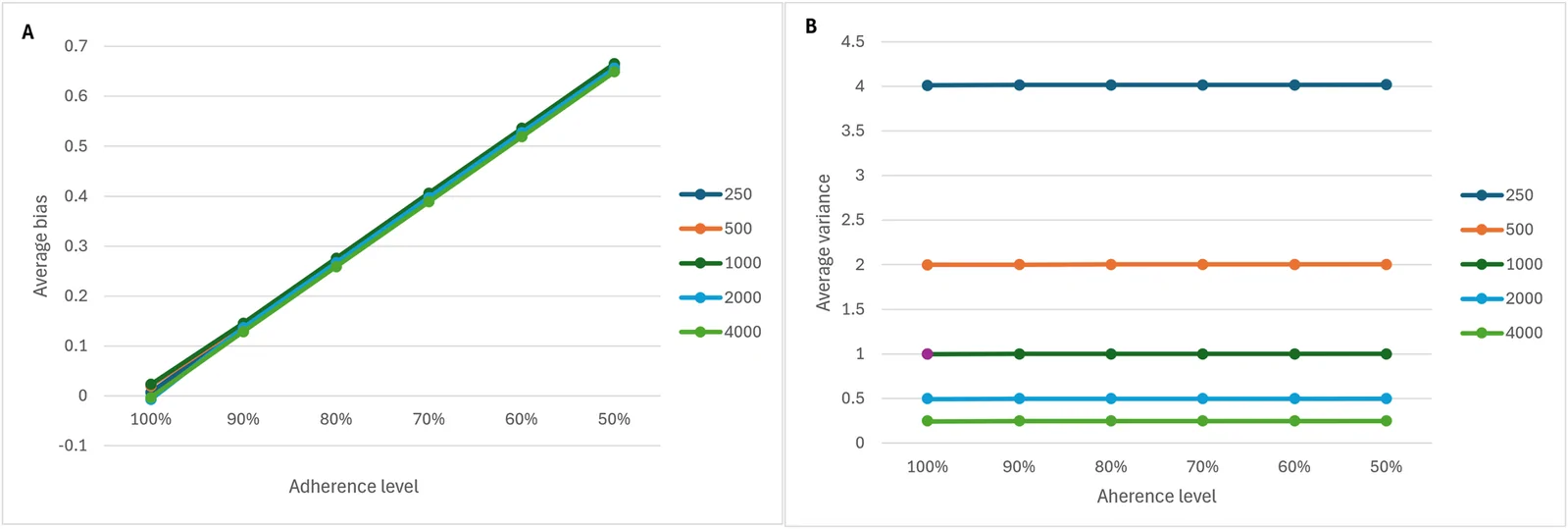
(A) Lower adherence but not sample size is associated with higher bias. (B) Smaller sample size but not adherence is associated with higher variance.

Next, we evaluated how the sample size and adherence level together influence the performance metrics by varying the sample size from 250 to 4000 participants at each adherence level. Our results (Figure 3A) demonstrate that at each sample size level, decreasing adherence level resulted in larger bias, and at each adherence level, sample size does not impact the mean bias. These results also demonstrated that even with a large sample size of 4000 participants (2000 in each arm), if adherence in the target population is low, the mean bias remains nearly as large as that observed with a smaller sample size. This suggests that increasing sample size alone does not eliminate the bias introduced by low adherence (Figure 3A). Although there is no effect of sample size on the mean bias, a smaller sample size is associated with larger variance of the bias in the effect estimate. For example, when the sample size is small with only 250 participants, the variance is 4; when the sample size increases to 4000, the variance is 0.25 (Figure 3B).

**FIGURE 4:**
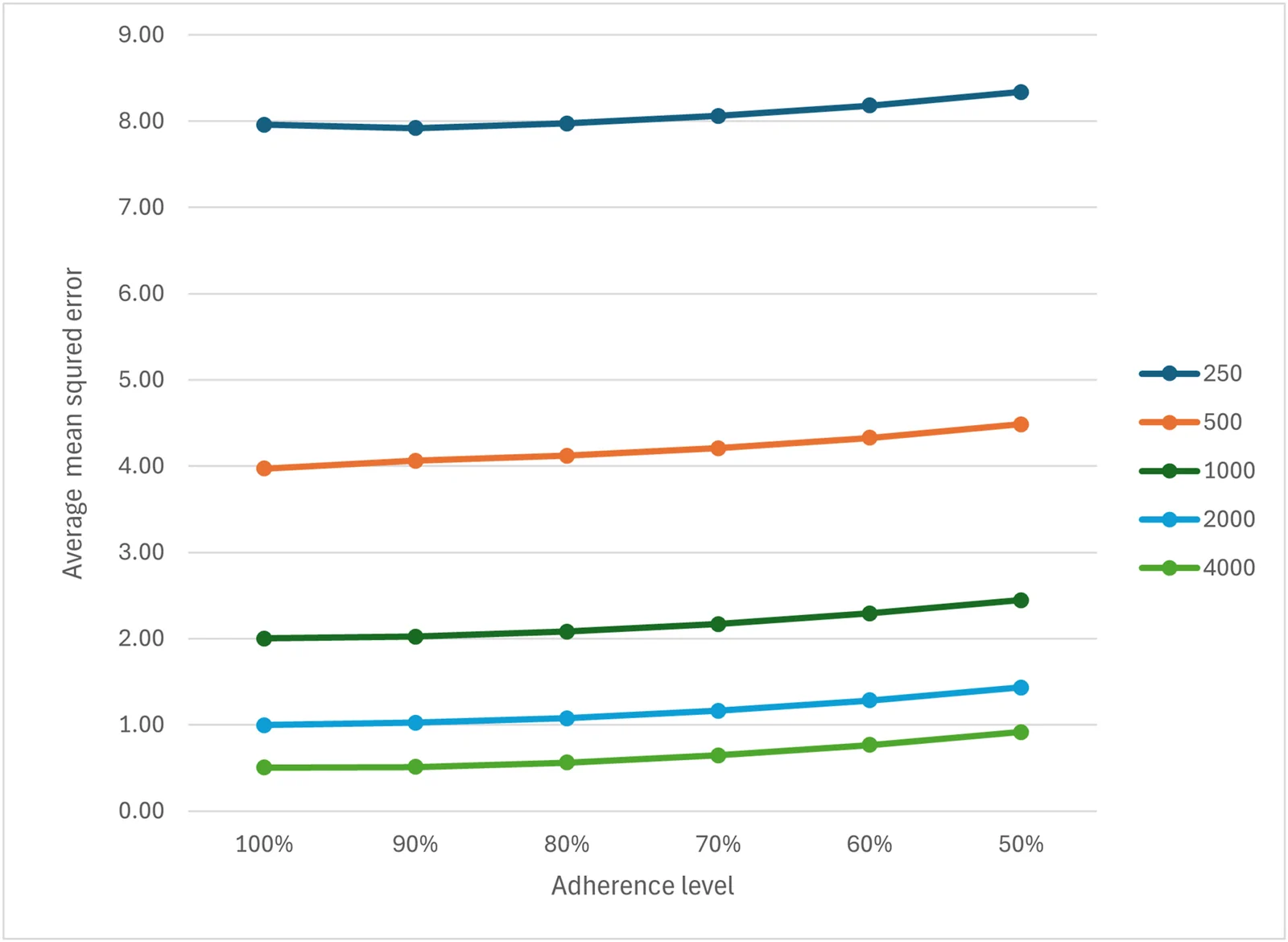
Mean squared error for various adherence levels and sample sizes.

Both sample size and adherence level impact mean squared error of the estimate. Sample size impacts the mean squared error through its influence on the empirical variance, whereas the adherence level influences the bias, and therefore, higher adherence and larger sample size are associated with a lower mean squared error (Figure 4). This result is expected because sample size impacts the variance of the estimate dramatically, and mean squared error is a function of variance and the square of bias.

## Discussion

In this simulation study, we found that the adherence of pregnant female participants to their prenatal MMS influences the bias and mean squared error of the effect estimate on GWG. Sample size does not impact the mean of bias, but it is inversely associated with the variation of bias. Even in the context of large sample size (*n* > 2000), low adherence leads to significant underestimation of the effect of prenatal MMS on GWG.

Our results indicated the importance of increasing and achieving high adherence to prenatal MMS among pregnant females to maximize the beneficial effect on GWG. Despite the consistently demonstrated efficacy of prenatal micronutrient supplements on pregnancy and birth outcomes, the implementation of an intervention remains challenging in LMICs. High cost and difficulties in distribution contribute to the lower coverage of the availability of this supplement to pregnant females. However, even in the area with higher coverage, adherence among females who have access to and receive prenatal micronutrient supplements is generally low [[14], [15], [16], [17]]. Using data from Demographic and Health Surveys from 22 LMICs, Sununtnasuk et al. [10] have shown that ∼81% of females received iron and folic acid supplements in these settings, but only 8% of them adhere to the recommended dose, which is defined as intake of ≥180 tablets during the entire pregnancy. A secondary data analysis from 35 sub-Saharan Africa countries demonstrated that >60% of pregnant females did not take their iron supplement for the recommended period [11]. Similar results were found in the studies from Ethiopia [17,18]. Adherence data from females who were provided MMS, which contains 15 micronutrients including 30 mg iron and 0.4 mg folic acid [19], are limited. In the previous meta-analysis of data from randomized controlled trials, we found that the average adherence (calculated as dividing the number of regimen pills consumed by the amount distributed for each female participant) ranged from 70% to 98% across trials [7]. A recent observational study from Indonesia found that 54% of the pregnant females started MMS intake in the first trimester. Of them, 75% consumed ≥90 tablets during the entire pregnancy [20]. Studies have found that females living in rural areas, from low-wealth households, with husband illiteracy and late antenatal clinical visits were associated with high nonadherence to prenatal micronutrient supplements [11,[21], [22], [23], [24]]. Therefore, in addition to increasing the coverage of prenatal micronutrient supplementation, future research should put more efforts to improve adherence at the individual level and account for residual confounding arising from factors that may be associated with both nonadherence and study outcomes. Potentially effective strategies that have been identified and used include education-based strategies, nutritional consultation, consumption monitoring by health workers or family members, and short message service reminders, but additional high-quality studies are urgently needed to examine the effectiveness and inform policies and intervention programs [25].

The intention-to-treat (ITT) approach has been widely accepted as the primary analytical approach for randomized nutritional intervention trials because it preserves the benefits of randomization and estimates the effect of assignment to an intervention. Under the ITT principle, all randomly assigned participants are analyzed according to their original assignment, regardless of their level of adherence. Consequently, the ITT estimate reflects the effectiveness of assigning an intervention in real-world settings, where incomplete adherence is common, rather than the efficacy of the intervention among participants who adhere to the protocol [26]. Our intent is not to suggest that the ITT approach is inappropriate for nutritional intervention studies. Rather, when ITT is used to evaluate and compare the efficacy of nutritional interventions, it is important to carefully maintain, assess, and report adherence, as varying levels of adherence may substantially influence the observed treatment effect.

The current simulation study has several strengths. First, it allows us to control both parameters, adherence levels, and sample sizes, to systematically evaluate their impact on bias in estimating the effect of prenatal MMS on GWG. These examinations are not feasible using real-world data because of the complex study design and the large sample size required. Furthermore, it is unethical to assign females into a lower level of adherence during their pregnancy in an intervention study design. Second, although all data are simulated data, they are created based on the results from a previous meta-analysis, in which we found that adherence level modifies the effect of MMS on GWG. By varying the percentage of females adhering to their prenatal MMS in the simulated data sets, we were able to examine how adherence level influences the effect that would be observed and calculate its bias from the true effect (a scenario when assuming all females adhere to their regimen, intaking ≥90% of their assigned regimen pills). Third, the results of our study help us further understand the critical role of adherence in nutritional intervention study in the context of different sample sizes. Our study demonstrated that the bias induced by low adherence cannot be compensated by a larger sample size; it is crucial to increase and maintain higher adherence in nutritional intervention studies among pregnant females. This result provides insights for future study design, resource allocation, implementation, and result explanation in nutritional research.

Limitations of our study need to be noted. First, adherence at the individual level was defined as a dichotomous variable rather than a continuous measure. Because existing trials generally reported high adherence levels and did not provide sufficient data on treatment effects across varying degrees of adherence, we were unable to simulate intervention effects at specific adherence levels. Consequently, adherence was defined as consumption of ≥90% of assigned supplements at the individual level. We acknowledge that this threshold is somewhat arbitrary and may result in the classification of participants with very similar adherence levels into different groups. However, the dose for each individual micronutrient included in prenatal nutritional supplements was based on existing studies and nutritional recommendations for pregnant females [6,27], and the goal for each individual pregnant female is to adhere to the supplement and finish the full or close to full dose during pregnancy. By varying adherence at the population level, our results are more consistent with the goal in the real world, provide data to inform nutritional supplementation programs, and research on efficient effort and resource distribution in pregnant females. Second, in this simulation study, we considered 2 parameters: the adherence level and sample size. There are other factors that could also influence the effect of prenatal nutritional supplementation on GWG, such as gestational age at which females start the supplementation, intervention duration, and maternal characteristics such as pre-existing health conditions or micronutrient insufficiency. These factors were beyond the scope of present simulations but may modify the intervention effect. Future studies incorporating these variables, together with validation using biomarker-based measures of micronutrient status and adherence, could provide a more comprehensive understanding of the conditions under which MMS is most effective [28]. Identifying females who are most likely to benefit from MMS is critical for maximizing the beneficial effects of nutritional intervention programs. Third, prenatal MMS is not a single, standardized intervention. Existing MMS may differ in nutrient composition, dosage, bioavailability, stability, and manufacturing quality, which could influence their efficacy. The present simulations assume a common intervention effect based on published prenatal MMS trials. Future simulation studies could evaluate how differences in MMS formulation influence adherence and efficacy.

Results from the current study will help researchers to optimize study design in the field of nutritional intervention and nutritional supplementation programs targeted for pregnant females, particularly in terms of ensuring sufficient sample sizes, monitoring and maintaining high adherence, and reporting adherence in publications and reports. Continuous efforts are guaranteed to identify barriers that prevent females from adhering to their prenatal nutritional supplements, explore factors that promote adherence [16,29]. Furthermore, our findings highlight the importance for future research to develop and validate innovative approaches to improve and maintain adherence to prenatal micronutrient supplements among pregnant females in LMICs to obtain accurate and reliable impacts of intervention programs, but more importantly to reduce risk of low birth weight and small-for-gestational-age birth of their infants.

## Author contributions

The authors’ responsibilities were as follows – EL: conducted the simulation, analyzed the data, and drafted the first manuscript; EW: critically read and revised the paper; and both authors: conceptualized the research question, developed the simulation plan, read, and approved the final manuscript.

## Data availability

The current study is a simulation study; the code to create the numerous simulated datasets is available upon request and with the permission of the administration of Boston Children's Hospital**.**

## Funding

The authors reported no funding received for this study.

## Declaration of generative AI and AI-assisted technologies in the writing process

The authors declare that no generative AI or AI-assisted technologies were used in the writing of this manuscript.

## Conflict of interest

The authors report no conflicts of interest.
